# Supplementary material for: Loss of HDAC11 ameliorates clinical symptoms in a multiple sclerosis mouse model
Source: Life Sci Alliance. 2018 Sep 24;1(5):e201800039. doi: 10.26508/lsa.201800039 (PMC6238389; doi:10.26508/lsa.201800039)
Supplement: Supplementary file 1 [file LSA-2018-00039_TableS1.docx]

**Table S1. PCR, qRT-PCR and ChIP Assay Primers**

**A. PCR primers in mouse genotyping**

| Gene | Forward Primer | Reverse Primer |
| --- | --- | --- |
| HDAC11 WT | tgctgcctgtgagccactgc | agaatggctgtctccctagg |
| HDAC11 KO | tgctgcCtgtgagccactgc | ccttggaatagcatctcagg |

**B. Real-Time qRT-PCR primers in gene expression analysis**

| Gene | Forward Primer | Reverse Primer |
| --- | --- | --- |
| HDAC1 | tgctggacttacgaaacagc | gtcgttgtagggcagctcat |
| HDAC2 | ctccacgggtggttcagt | cccaattgacagccatatca |
| HDAC3 | ttcaacgtgggtgatgactg | ttagctgtgttgctccttgc |
| HDAC4 | cacacctcttggagggtacaa | agcccatcagctgttttgtc |
| HDAC5 | gagtccagtgctggttacaaaa | tacacctggaggggctgtaa |
| HDAC6 | gaaggaggagctgatgttgg | tcatgtactgggttgtctccat |
| HDAC7 | gccttgagagaacagtcca | ccaagggctcaagagttctg |
| HDAC8 | aggtgatgaggaccatccag | accctccagaccagttgatg |
| HDAC9 | gtgcagccagatggagtgg | ggcccataggaacctctgat |
| HDAC10 | ttccaggatgaggatcttgc | acatccaatgttgctgctgt |
| HDAC11 | GCGCTACAACATCACCTTCA | TTAAGATAGCGCCTCGTGTG |
| HPRT | tgctcgagatgtcatgaagg | tatgtcccccgttgactgat |
| CCL2 | GTCTGTGCTGACCCCAAGAAG | TGGTTCCGATCCAGGTTTTTA |
| CCL2 (human) | AAGATCTCAGTGCAGAGGCTCG | TTGCTTGTCCAGGTGGTCCAT |
| CD4 | GTCTCTGAGGAGCAGAAAGTAG | CTGGAGTCCATCTTGACCTTATC |
| CD11b | GACCGTCTGCGCGAAGGAGATA | CGCCTGCGTGTGTTGTTCTTTG |
| CD8 β-chain | CATCCTGCTTCTGCTGGCATT | TGGGCGCTGATCATTTGTGAAA |
| GAPDH | AGCTTGTCATCAACGGGAAG | TTTGATGTTAGTGGGGTCTCG |

**C. qPCR primers in ChIP assays**

| Gene | Forward Primer | Reverse Primer |
| --- | --- | --- |
| CCL2 | CGAGGGCTCTGCACTTACTC | AGTGAGAGTTGGCTGGTGCT |
